# Editorial: International Myopia Institute White Paper Series 2023

**DOI:** 10.1167/iovs.64.6.1

**Published:** 2023-05-01

**Authors:** Nina Tahhan, James S. Wolffsohn, Padmaja Sankaridurg, Jost B. Jonas, Mark A. Bullimore, Ian Flitcroft, Lisa A. Ostrin, Christine Wildsoet, Serge Resnikoff

**Affiliations:** 1 Brien Holden Vision Institute, Sydney Australia; 2 School of Optometry and Vision Science, Faculty of Medicine, University of New South Wales, Sydney, New South Wales, Australia; 3 College of Health & Life Sciences, Aston University, Birmingham, United Kingdom; 4 Department of Ophthalmology, Medical Faculty Mannheim, Heidelberg University, Mannheim, Germany; 5 Institute of Molecular and Clinical Ophthalmology Basel, Basel, Switzerland; 6 University of Houston, College of Optometry, Houston, Texas, United States; 7 Centre for Eye Research Ireland, School of Physics and Clinical and Optometric Sciences, Technological University Dublin, Dublin, Ireland; 8 Department of Ophthalmology, Children's Health Ireland at Temple Street Hospital, Dublin, Ireland; 9 UC Berkeley Wertheim School Optometry & Vision Science, Berkeley, California, United States

The first series of International Myopia Institute (IMI) white papers was published early in 2019 in response to the growing need for consensus and clinical management guidance on the ever growing, and sometimes conflicting, evidence base around myopia development and management. The IMI successfully brought together leading academics, clinicians, industry, and other stakeholders to address areas that were agreed to be of importance. Together, more than 85 multidisciplinary experts generously dedicated their time and resources to produce the first series of white papers. In addition to defining and summarizing evidence from experimental animal models, human trials, genetics studies, and the breakthrough technologies of the time for slowing myopia, the white papers identified directions for future research, also establishing definitions and clinical guidelines for application in both myopia-related research and its clinical management, thereby providing researchers and clinicians with both a common language and unified set of goals.^1^^–^^8^

A second series of IMI white papers released in 2021 considered and synthesized other areas of significance, including pathologic myopia, the impact of myopia, risk factors for myopia, roles of accommodation and binocular vision in myopia development and progression, and the prevention of myopia and its progression.^9^^–^^14^ In addition, a digest that provided updates on topics covered in the 2019 papers was published.^15^

This third series of white papers published in 2023 highlights other key areas of myopia research and management, namely, evidence around onset, progression, and management of myopia in the less typical young adult^16^ and pediatric (infant and pre-school children less than 5 years of age)^17^ populations, the role of the choroid in eye growth control and myopia management,^18^ and a detailed anatomic paper on the “non-pathological” ocular tissue changes observed in moderate to high myopia.^19^ Emerging evidence for roles of the choroid in both myopia development and myopia control warrants further attention, particularly for clinicians who may be grappling to understand how research findings might translate to clinical practice. A thorough characterization of non-pathological ocular changes in myopia may help researchers to further elucidate the mechanism of axial elongation and better understand associated secondary pathologies. Also included in this series is a report on the results of an international survey of practitioners on myopia management attitudes and strategies in clinical practice.^20^ This paper reflects on how practices and attitudes regarding myopia management may have changed over the past decade based on other similar, previously published survey results.^21^^,^^22^ The latest results indicate that single vision spectacles and contact lenses are still the most prescribed methods of correction, although clinical activities related to myopia management, including the prescription of myopia control devices and therapies, appear to be increasing. More needs to be done to establish myopia control as the standard of care for progressive myopia around the world.

To help stakeholders keep up to date with this fast moving field, new findings across some of the key topics in myopia research since the 2019 digest have been reviewed by experts and summarized as the IMI 2023 digest.^23^ Consolidation, consensus, and updates on all the latest evidence in the form of these white papers is an important resource for practicing clinicians who may not have the time and resources available to sift through the ever evolving and growing body of evidence to understand how the most recent findings translate to clinical practice and how to implement the most appropriate and effective treatment strategies.

All IMI articles and associated infographics, freely available at https://myopiainstitute.org/, serve as tools to help with this process. By highlighting gaps in our current knowledge, they also provide a guide for ongoing and future research. World wide web analytics from the IMI online platform show year on year growth in the number of members, increased new and returning visitors, and greater views and downloads of clinical summaries, white papers, and infographics. The referral pathway, or traffic generated from links provided by third parties, has also grown by over 200% in the past year, indicating the increasing value of the website's resources in the wider online discussion of myopia. With clinical summaries translated into 15 languages, the platform strives to maintain a truly global audience, as indicated by increased views and downloads in over 187 different countries.

By 2050, it is predicted that almost half of the global population will be myopic, with 10% at levels worse than −5.00 diopters^24^ and hence at greater risk of sight-threatening complications and visual impairment.^25^^–^^27^ Every diopter matters^28^ and hence every clinician should be supported and encouraged to introduce evidence-based myopia management to improve the quality of life and well-being of their patients, their families, communities, and the broader society.^13^ We commend all those who are striving to make this change and thank all those who have contributed to these efforts. We also invite all who are willing and interested to join the IMI in these efforts.
